# Magnitude of early relaparotomy and its outcome among patients who underwent laparotomy in a tertiary hospital in Eastern Ethiopia: a cross- sectional study

**DOI:** 10.1186/s12893-024-02338-x

**Published:** 2024-02-09

**Authors:** Eyobel Amentie, Badhaasaa Beyene, Mekonnen Sisay, Muluwas Amentie Zelka, Shambel Nigussie

**Affiliations:** 1 School of Medicine, College of Health and Medical Sciences, Haramaya University, Harar, Ethiopia; 2 School of Pharmacy, College of Health and Medical Sciences, Haramaya University, Harar, Ethiopia; 3 Department of Public Health, College of Health Sciences, Assosa University, Assosa, Ethiopia

**Keywords:** Relaparotomy, Indication, Outcome, Eastern Ethiopia

## Abstract

**Introduction:**

Several studies conducted worldwide revealed the magnitude of early relaparotomy and its outcome among patients undergoing laparotomy. However, there was very little evidence on the magnitude of early relaparotomy and its outcome among patients who underwent laparotomy in Ethiopia, especially in the study area.

**Objective:**

this study aimed to the assess magnitude of early relaparotomy and its outcome among patients who underwent laparotomy in a Tertiary Hospital in Eastern Ethiopia.

**Methods:**

A retrospective cross-sectional study was conducted. All patients who underwent laparotomy during the data retrieval period were included. Data were collected using a data abstraction checklist from patients’ medical records. The collected data were entered, cleaned, and analyzed by using SPSS version 23. Descriptives statistics were generated where by continuous variables were summarized into means and standard deviation and categorical variables were summarized as the frequency with proportions.

**Result:**

The magnitude of relaparotomy was 6.8%. Among 82 patients included in the final analysis, 53 (64.6%) were males and the mean (± SD) age of patients was 33.32 ± 16.63 years. The major indications for relaparotomy were intra-abdominal collection (26.8%) and anastomotic leak (24.4%). Among 82 patients who underwent relaparotomy, 52(63.4%) were developed post relaparotomy complications, and 30(36.6%) patients died.

**Conclusion:**

The magnitude of early relaparotomy was 6.8%. The magnitude of in-hospital mortality was high in comparison to earlier study findings from developing countries. About three fourth of patients who underwent relaparotomy were developed postoperative complications.

## Introduction

Relaparotomy is re-operative abdominal surgery carried out during the postoperative period after laparotomy and causally related to the first operation within the first 60 days of the primary operation. It is considered as the access to the abdominal cavity before the complete healing of the surgical wound from a previous operation [1–3].

Relaparotomies can be classified as early,ate, urgent (emergency), elective, planned, unplanned, radical, and palliative depending on the time of reoperation after initial surgery, nature of urgency, and goal of reoperation [4]. Early relaparotomy is relaparotomy which is done within the first 30 days of initial surgery while late relaparotomy performed between 30 and 60 days of initial surgery operation [1–3].

Relaparotomy is a lifesaving procedure for patients who develop complications following elective or emergency laparotomy. Recognizing complications of postoperative peritonitis that developed early after intra-abdominal surgery and performing relaparotomy to eliminate any pathogens immediately can be lifesaving [5–7]. However, undertaking proper precautions to ensure proper hemostasis and asepsis can go a long way in decreasing the incidence of relaparotomy. Taking a calculative decision before embarking on a hasty decision of relaparotomy is an important mechanism to decrease the incidence of relaparotomy [7].

Worldwide, some studies found that the magnitude of relaparotomy ranged from 0.5–15% [3, 6] as well as mortality after relaparotomy ranged from 24 to 71% which is associated with different factors such as elderly patients, peritonitis at the initial surgery, multiorgan failure, multiple relaparotomy and anastomotic leak [6]. An observational study of relaparotomy in Western India showed the mortality rate was 34.72% with the most common reason for mortality was septicemia [6] (Patel et al., 2017). Another study done at AOU Policlinic University of Catania also resulted that the mortality rate after relaparotomy was found to be 38.7% with most common reason for mortality was sepsis and multi-organ failure [8]. The mortality rate of patients after relaparotomy in the studies done Africa such as South Africa, Congo, Tanzania and Ethiopia (at St Paul Hospital) showed 14%, 17.6%, 39.6% and 14.7% respectively with the most common reason for death were sepsis, septic shock and multi organ failure [9–12]. .Even if limited studies conducted previous worldwide, there was very little evidence on the magnitude of early relaparotomy and its outcome among patients who underwent laparotomy in Ethiopia, especially in the study area. So, this study aimed to the assess magnitude of early relaparotomy and its outcome among patients who underwent laparotomy in a Tertiary Hospital in Eastern Ethiopia.

## Methods and materials

### Study area, design and period

Across-sectional study was conducted at Hiwot Fana Comprehensive Specialized Hospital (HFCSH) which found in Harar regional state, Eastern Ethiopia. HFCSH is one of the two government Hospitals in Harar and its administration was totally taken by Haramaya University as teaching Hospital. It has been serving as the Referral Hospital for the populations found in Harari region, east and west Harerge zones, Dire Dawa federal town administrative city and Jigjiga town which was the capital city of Somali regional state. HFSUH has different departments. Surgery department was one of the major departments of the hospital. The surgical ward has 52 beds, intensive care unit and three major operation theatres. The data was collected from August 1 to September 30, 2020.

### Source and study population

All patients who underwent laparotomy at HFCSH Surgery ward from September 1, 2016 to August 30, 2020 were considered as source population. All patients who underwent relaparotomy during retrieval period considered as study population.

### Eligibility criteria, sample size and data collection procedure

All patients who underwent laparotomy and relaparotomy at HFCSH Surgery ward from September 1, 2016 to August 30, 2020 were included. Patients who had incomplete patient records, patients discharged against relaparotomy, those not returning to the study setting after laparotomy, those who need relaparotomy but died before re-operation, and referred from other Hospitals for relaparotomy were excluded. There was a total of 92 relaparotomy cases from September 1, 2016 to August 30, 2020, of which 82 cases had complete data, and were included in the study. Data were collected using pre-tested check list and collected by trained nurses and medical residents. Data sources were operation room log books, patients’ record books and patients’ charts. First the total number of patients who underwent laparotomy were counted from operation logbook and their charts were retrieved from card room. From these patients those who needed relaparotomy were identified. Then after, the necessary information was gathered specifically on patients’ who underwent relaparotomy from respective medical record log books and patients charts to determine outcome of relaparotomy. As a result, detail data was collected on the socio-demographic variables, diagnosis at initial laparotomy, duration of illness before arrival, indications of relaparotomy and outcome of patients after relaparotomy were recorded.

Before starting the data collection, data collection checklist was pre-test at Jugol General Hospital and two-day training for data collectors and supervisors were given. to ensure quality of data. Furthermore, supervisors were checking collected data every day then provide feedback and made necessary correction during data collection. Finally, completeness, accuracy, and clarity of the collected data were checked carefully by supervisors and principal investigators.

### Operational definitions

#### Early relaparotomy

Relaparotomy done in the first 30days of primary operation (Tihitena et al., 2018).

#### Outcome of relaparotomy

In this study the outcome of relaparotomy could be developing post relaparotomy complications and death.

#### Post relaparotomy complications

In this study patients who developed the following complications after relaparotomy considered as post relaparotomy complications: hospital acquired infections, septic shock, Fluid and electrolyte imbalance, anemia, malnutrition, multi organ failure and had an indication of second relaparotomy.

#### Hospital acquired infections

In this study if the patients developed any new infection after relaparotomy considered as had hospital acquired infection.

#### Septic shock and multiorgan failure

In this study if the patient developed septic shock but not fulfilled criteria for the multiorgan failure considered as had septic shock while patients progressed to multiorgan failure considered as had multiorgan failure.

### Data processing and analysis

Data were checked for completeness and entered to SPSS version 23 for further analysis. The. Descriptives statistics were generated where by continuous variables were summarized into means and standard deviation and categorical variables were summarized as the frequency with proportions.

### Ethical consideration

Before beginning data collection, ethical clearance was secured from Institutional Health Research Ethics Review Committee (IHRERC) of the College of Health and Medical Sciences, Haramaya University (Ref number = IHRERC/151/2020). Letter of permission was obtained from College of Health and Medical Sciences, School of Medicine to the study area. The patients’ identity like name was not stated in the questionnaire and confidentiality and privacy of the patients’ information was kept.

## Results

### Socio -demographic and clinical characteristics

In this study laparotomy was done for 1335 patients. Of those, relaparotomy was done only for 91 patients (6.81%) and 82 patients had complete data. Among 82 patients included in the final analysis, 53 (64.6%) were males and the mean (± SD) age of patients was 33.32 + 16.63 years. The most common diagnosis at initial surgery was viscous perforations which accounted for 25.6% of the cases and followed by Gangrenous Bowel Obstruction 20(24.4%). Around 76(92.7%) of patients underwent relaparotomy were at the emergency base (Table 1). Among patients who underwent relaparotomy, the common types of surgery done at a primary laparotomy were resection and anastomosis of small bowel 23(28%) and Omental patch 16(19.5%) (Table 2). The other types of surgery done at a primary laparotomy were colostomy 8(9.8%), appendectomy and lavage 8(9.8%), simple bowel repair 5(6.1%), ileostomy 3(3.7%), stoma reversal 3(3.7%), damage control surgery 3(3.7%), small bowel adhesion release 3(3.7%), resection and anastomosis of large bowel 2(2.4%), peritoneal lavage only 2(2.4%), derotation of small bowel volvulus 2(2.4%), simple exploration 1(1.2%), packing liver injury 2(2.4%) and mass excision 1(1.2%).

**Table 1:** Socio demographic and some clinical characteristics of patients who underwent early relaparotomy at HFCSH Surgery ward, Harar, Ethiopia, September 2016 to August 2020

Characteristics		Frequency (%)
**Sex** (*N* = 82)	Male	53 (64.6)
	Female	29 (35.4)
Mean and Standard Deviation of Age (years)	33.32 ± 16.63	
**Age** (*N* = 82)	< 18 years	9 (10.9)
	18 to 36 years	31 (37.8)
	> 37 years	28 (34.1)
**Diagnosis at primary laparotomy (***N* = 82**)**	Gangrenous Bowel Obstruction	20 (24.4)
	Viscous perforations	21 (25.6)
	Abdominal trauma	16 (19.5)
	Perforated Appendix	8 (9.8)
	Other GI and pancreatic Surgery	14 (17.1)
	Stoma (Ileostomy or Colostomy) reversal	3 (3.7)
**Types of relaparotomy**	Emergency	76 (92.7)
	Planned	6 (7.3)

**Table 2:** Common post-operative complications and causes of death among patients who underwent early relaparotomy at HFCSH, Surgery ward, Harar, Ethiopia, September 2016 to August 2020

Post-operative Complications	Frequency	Percentage
Hospital acquired infections	54	88.5
Septic shock	31	73.8
Fluid and electrolyte imbalance	29	47.5
Anemia	25	41
Malnutrition	23	37.7
Multi organ failure	14	22.9
**Causes of Death**		
Postoperative Septic shock	16	53.3
Multi-organ failure	12	40
Ongoing Bleeding	2	6.7

The major indications for relaparotomy were intra-abdominal collection 22(26.8%), anastomotic leak 20(24.4%), Omental patch failure11(13. 4%), and Stoma complications 9(11%). The common types of surgery done at relaparotomy were peritoneal lavage only 24(29.3%), re-anastomosis or primary repair small bowel perforation 15(18.3%), ileostomy 12(14.6%), and refashioning of stoma 9(11%).

### Magnitude of relaparotomy and its outcome

The magnitude of relaparotomy was 6.8%. Of 82 relaparotomy cases, 52(63.4%) developed different types of complications. The predominant postoperative complications were hospital acquired infections, septic shock, and fluid and electrolyte imbalance. Among 82 relaparotomy cases, 30 (36.6%) patients died and the underlying causes of death were septic shock 16 (53.3%), multi-organ failure 12(40%), and ongoing bleeding 2(6.7%) (Table 2).

## Discussion

This study aimed to the assess magnitude of early relaparotomy and its outcome among patients who underwent laparotomy in a Tertiary Hospital in Eastern Ethiopia.

The mean age of the patients in this study who underwent early relaparotomy was 33.3 years, which is comparable to other studies’ findings from tertiary hospitals in India and Africa [6, 9–12]. According to the results of this study, the magnitude of early relaparotomy was 6.8% which is consistent with earlier research findings from St Paul hospital (6.9%) and Tanzania (7.6%) [10, 11].

In current study, the index surgery done at initial laparotomy was bowel resection and anastomosis in 28% of patients and followed by omental patch for perforated peptic ulcer disease in 19.5% patients. This finding is comparable with study report done in Tanzania [10] but differ from studies carried out in Ethiopia, South Africa and Congo as they revealed appendectomy for perforated appendicitis was the leading index surgery that required relaparotomy [3, 9, 11, 12]. This difference could be due to variation of numbers of years the patient’s data had been taken. Three years data were taken from Ethiopia and one and half years data were taken from South Africa and Congo.

In this finding, the common indications for relaparotomy were intra-abdominal collection (26.8%) and anastomotic leak (24.4%.) This was almost comparable with other studies done in Africa including Ethiopia and India [9, 13, 14]. In contrast this, some studies done in United Kingdom, Poland and Turkey revealed hemorrhage as the commonest indication of relaparotomy [5, 14, 15] .This discrepancy might be explained by the complexity and type of the first surgery as there is high burden of liver and pancreatic surgery, colorectal surgeries and trauma which were usually complicated by bleeding.

The finding of the current study showed that 74.4% of patients developed different types of complications after relaparotomy. The common post-relaparotomy complications were hospital acquired infection (88.5%) and septic shock (73.8%). This finding was consistent studies done in South Africa, Tanzania, India, UK and Ethiopia (at St Paul Hospital) [10, 11, 16].

The mortality rate of relaparotomy patients in this study was 36.6% which is higher than the study results report from St Paul Hospital, South Africa, Congo and India ranged from 14 to 21% [5, 9, 12]. This discrepancy may due to the difference in the presence of complication. This study found that septic shock and multiorgan failure were being the commonest reason of death which similar to previous studies findings [3, 8, 17].

## Conclusion

In this study the magnitude of early relaparotomy was 6.8% which is comparable with earlier research findings. The common types of surgery done at relaparotomy were peritoneal lavage only, re-anastomosis or primary repair small bowel perforation, ileostomy, and refashioning of stoma. About three fourth of patients who underwent relaparotomy were developed postoperative complications. The in-hospital mortality rate after relaparotomy was high.

## Data Availability

The datasets used and/or analyzed during the current study are available from the corresponding author on reasonable request.
